# Flowering Newsletter 2023

**DOI:** 10.1093/jxb/erad258

**Published:** 2023-07-07

**Authors:** Rainer Melzer

**Affiliations:** Flowering Newsletter Editor, JXB; School of Biology and Environmental Science and Earth Institute, University College Dublin, Ireland

**Keywords:** Apomixis, auxin, carpel, crops, ethylene, flowering time, fruits, seeds, senescence, sperm cells

**The word ‘fruit’ is derived from the latin ‘fructus’ which itself is said to be derived from ‘frui’, which means to enjoy. Along those lines, I hope this year’s Flowering Newsletter brings a lot of joy, because fruits and seeds feature in multiple articles.**

There would of course be no fruits without fertilization. Indeed, fertilization in flowering plants is an intricate process, involving pollen tube growth and the formation of a triploid endosperm. Sperm cells play an important part in this process, and Flores-Tornero and Becker (2023) provide an overview over the last 50 years of research on sperm cell isolation. They outline how far we have come since the first isolation of sperm cells in 1973 (Cass, 1973): sperm cells can now be isolated and analysed in great detail to answer questions related to their biochemical, transcriptional, and epigenetic status.

Before fertilization takes place, the pollen tube has to grow all the way down from the stigma to the ovule. This is required because in flowering plants the ovule is protected by one of the most fascinating evolutionary novelties, the carpel. Reyes-Olalde *et al.* (2023) explore the complex processes involved in carpel development, and also discuss the evolutionary origin of the carpel.

Of course, one reason why the carpel receives so much attention is that it develops into the fruit, and fruits feed the world. From an agricultural perspective, maximizing fruit number—and thus yield—is desirable. However, as Sadka *et al.* (2023) explain, plants have evolved several feedback loops that finely regulate how much fruit is produced. While those feedback mechanisms might be an important evolutionary strategy to ensure reproductive success in unpredictable environments, breaking fruit-limiting feedback loops might be a promising avenue for plant breeding to increase yield (Sadka *et al.*, 2023).

While Sadka *et al.* (2023) provide insights into feedback mechanisms regulating fruit set, Balanzà *et al.* (2023) approach the topic of how the end of flowering is regulated from a molecular genetic perspective. They outline the numerous genes and pathways involved in this process in Arabidopsis. Both articles agree that phytohormones, in particular auxin, play a major role in controlling reproductive arrest and fruit numbers.

The articles by Balanzà *et al.* (2023) and Sadka *et al.* (2023) show that we are only beginning to understand the molecular mechanisms governing the end of flowering. In contrast, the beginning of flowering has been among the first traits dissected genetically in plants. However, the wealth of data also bears the potential for confusion. One of those potential sources of confusion is related to the genetic control of photoperiod insensitivity in wheat and barley. Slafer *et al.* (2023) dissect this topic in detail and illustrate how mutations in orthologous genes can yield seemingly opposite effects in terms of photoperiod insensitivity in those two closely related cereals. The article shows that careful analysis of molecular mechanisms and phenotypic responses is at the heart of interpreting gene regulatory networks, and that insights into molecular mechanisms can be useful for cereal breeding (Slafer *et al.*, 2023).

I stated above that there are no fruits without fertilization, but of course, as always in biology, there are many exceptions to this rule. A particularly interesting one is apomixis, which is explored in detail by Cornaro *et al.* (2023). Apomixis is the asexual production of seeds by parthenogenesis. As Cornaro *et al.* (2023) outline, this is a topic that has been studied for well over 100 years and is understood in great detail at the cytological level. Apomixis bears a huge potential for agriculture, as it offers the opportunity to produce seeds and fruits that are genetically identical to the mother plant (Cornaro *et al.*, 2023). It will be interesting to see if this potential can be exploited for our main staple crops in the future.

Seeds and fruits feed the world, yet the beauty of flowers lies in their magnificent petals. Vivian Irish (2023) provides a personal account of her research on petal cell shape. Her article is also a lesson of how grand questions in biology—the formation of tissues and organs—are approached experimentally, and the power which lies in efficient mutagenesis screens. Even more intriguing, she shows that with every answer we open a box to a new set of questions. One hypothesis that emerges is that the chirality of molecules is linked to the chirality of macroscopic structures (Irish, 2023).

As beautiful as petals are, their life also comes to an end. Not surprisingly though, this is a highly orchestrated process that involves numerous genes. Somewhat fittingly, the volatile beauty of petals is mediated by the volatile phytohormone ethylene (Parveen *et al.*, 2023). As outlined by Parveen *et al.* (2023), a better understanding of floral senescence may also eventually lead to enhanced crop yields.

This issue of the Flowering Newsletter is a testament that discoveries related to fruits and seeds, petals, and flowering time continue to push the boundaries of our knowledge (Chapman, 2022; Fattorini and Ó’Maoiléidigh, 2022; Steinbrecher and Leubner-Metzger, 2022). However, what is sometimes also astonishing is how little we still know about some phenomena. How does flowering end? How does a plant regulate the number of fruits it produces? We are beginning to make strides on those questions in a few selected species, but much more needs to be learned. This relates particularly to crop plants. It will be interesting to see whether and how some of the observations outlined in this issue of the Flowering Newsletter translate from model plants such as Arabidopsis to the diversity of crops grown for human consumption, and whether new discoveries can be used to increase crop performance. This may relate to testing new allelic combinations, as suggested for flowering time in cereals (Slafer *et al.*, 2023), but may also involve exploiting the plethora of effects of phytohormones. Indeed, phytohormones are involved in virtually all developmental processes, and carpel, seed, and fruit development or senescence are no exception here (Balanzà *et al.*, 2023; Parveen *et al.*, 2023; Reyes-Olalde *et al.*, 2023; Sadka *et al.*, 2023). Phytohormones or derivatives thereof are already used in agriculture to control, for example, plant height, and we may we be able to use similar compounds in a targeted manner in the future to further increase crop performance.

Crop breeding and crop management have been enormously successful in increasing crop performance in the past decades. New discoveries are waiting to be made and I am convinced that they will contribute to further improving agricultural practices and generating new crop varieties. New research topics on seeds and fruits will mature in the years to come. Likewise, I sincerely hope the seeds of joy for research on flowers have been firmly planted with this issue of the Flowering Newsletter.
